# The transition of human resources for health information systems from the MDGs into the SDGs and the post-pandemic era: reviewing the evidence from 2000 to 2022

**DOI:** 10.1186/s12960-023-00880-y

**Published:** 2023-12-01

**Authors:** Pamela A. McQuide, Andrew N. Brown, Khassoum Diallo, Amani Siyam

**Affiliations:** 1 Global Health Workforce Consultant, IntraHealth International, 6340 Quadrangle Drive, Suite 200, Chapel Hill, United States of America; 2 Global Health Workforce Consultant, Canberra, Australia; 3 Coordinator Data, Evidence and Knowledge Management UHL Division, World Health Organization, Geneva, Switzerland; 4 Health Information System, Regional Office for South-East Asia, World Health Organization, Geneva, Switzerland

**Keywords:** HRH, HRHIS, NHWA, WISN, Health workforce, Digital health, DHIS2

## Abstract

**Background:**

This review paper offers a policy-tracing trend analysis of national experiences among low- and middle-income countries in strengthening human resources for health information systems (HRHIS). This paper draws on evidence from the last two decades and applies a modified Bardach’s policy analysis framework. A timely review of the evidence on HRHIS and underlying data systems is needed now more than ever, given the halfway mark of the Global Strategy on Human Resources for Health: Workforce 2030 and the protracted COVID-19 pandemic and other global health emergencies, over and above the increasing need for health and care workers to provide essential health services.

**Main text:**

Considering World Health Assembly resolutions and HRH-related global developments between 2000 and 2022, we targeted peer-reviewed and gray literature covering the inception, impact, bottlenecks, and gaps of HRHIS. We also considered results from a Bill and Melinda Gates Foundation-funded project that assessed HRH data systems in 21 countries and the use of HRH data and information for policy, planning, and management. Aligned with the National Health Workforce Accounts (NHWA), we identify priority themes related to digital priorities for HRHIS and governance/leadership and present case studies of five countries that pursued different pathways to successfully develop their HRHIS.

**Summary conclusion:**

Over the last two decades, considerable progress has been achieved through a scaled-up implementation of HRHIS combined with the skills needed to analyze and use data, sustain systems functionality, and make systematic improvements over time. Global health development aid investments and technical innovations have led to advancements in HRHIS, district health information software (DHIS2), and partner collaborations during the HIV/AIDS, Ebola, and COVID-19 crises. Although the progressive implementation of NHWA continues to steer country-level efforts through standardized indicators and regular reporting, traditional challenges remain, such as data systems fragmentation, lack of interoperability between systems, and underutilization of reported data. Encouragingly, some countries demonstrate strong governance and leadership capacities and others strong HRHIS digital capacities. Both HRH and health service data are needed to inform on-demand decisions during times of emergencies and pandemics as well as during routine essential health services delivery. Evidence-based examples from distinctive countries demonstrate that reliable HRHIS is achievable for better planning and management of the health and care workforce.

## Introduction

Prior to 2000, human resources for health (HRH) data were scarce, incomplete, and fragmented and their value undermined especially as health workers were generally perceived as recurrent costs to health systems rather than as a strategic input that must be enumerated, remunerated, and retained. The Joint Learning Initiative (JLI), a global workforce assessment conducted by over 100 health leaders, called on national and global leaders and funders to prioritize the health worker as an asset and HRH systems as a necessity—in particular, the development and analysis of quality data and human resources for health information systems (HRHIS) on all health workers, including their social attributes and work functions, and inclusive of all those who are immigrating into and emigrating out of individual countries. JLI called on national and international organizations to “enhance their investment in information and knowledge on human resources…those investments would provide a global public good” [1].

Over the past two decades, three global health crises have amplified the need for quality and timely HRH data and information—the HIV/AIDS and Ebola epidemics and the COVID-19 pandemic—triggering the creation of new national and international funding streams. This paper differs from previous HRHIS published literature using a policy analysis framework to introduce and consider significant country-level HRHIS reviews, a 2020 study funded by the Bill and Melinda Gates Foundation, global investments from donors, and experiences from selected case studies from World Health Organization (WHO) regions.

The specific objective of this paper is to provide a policy-tracing analysis of the key events, strategies, innovations, good practices, bottlenecks, challenges, and lessons learned in implementing HRHIS, specifically in low- and middle-income countries (LMICs), and the interlinkage with the global implementation of the National Health Workforce Accounts (NHWA).

## Methods

We applied a modified Bardach’s policy analysis framework [2, 3] to identify the key policy processes and trends in the implementation of HRHIS over the past 20 years. The steps undertaken were to (1) define the primary objective of the study; (2) assemble the evidence; (3) construct and demonstrate the approaches and alternatives followed in developing and strengthening HRHIS; (4) select a common criteria to examine and compare the evidence; (5) project the principal outcomes (through a comparison of country case studies and experiences); (6) confront the trade-offs and lessons learned (gleaned from country experiences); (7) synthesize the evidence; and (8) conclude with key findings, major influences, and future outlook.

### Step 1: Strengthening HRH data and information systems—a global mandate and strategic objective

In 2006, World Health Assembly (WHA) resolutions passed by WHO member states called on countries to build quality, evidence-based information systems for HRH. These resolutions and other commitments offered strategic and operational directives to attaining credible, current, and reliable health and HRH data and information to guide planning, management, and policies. Table 1 provides a selective listing of key WHA resolutions that have had global impact in strengthening HRHIS in WHO member states.

**Table 1:** Key World Health Assembly resolutions impacting the global development and use of HRHIS (2000–2022)

Date	Resolution	Importance
May 2006	**WHA 59.27** Strengthening nursing and midwifery [4]	Focused on the global shortage of doctors, nurses, and midwives; similar timing as the World Health Report (2006)
May 2007	**WHA 60.27** Strengthening of health information systems [5]	Encouraged WHO member states to develop and use accurate data to estimate workload for health workers
May 2010	**WHA 63.15** Health worker information systems and** WHA 63.16** Global Code of Practice on International Recruitment of Health Personnel [6]	Encouraged WHO member states to develop health worker information systems including data on migration of health personnel and encouraged member states to promote sustainable health systems
May 2016	**WHA 69.19** Global Strategy on HRH: Workforce 2030 [7]	Instrumental in laying out four major initiatives: having evidence-informed policies to optimize the workforce; catalyzing investments in health labor markets to meet population needs; building institutional capacity and partnerships in HRH governance and leadership; and using data for monitoring and accountability, including the implementation of the NHWA and annual reporting to the WHO Global Health Observatory
May 2017	**WHA 70.6** Working for Health: 5-year action plan for health employment and inclusive economic growth (2017–2021) [8]	A mechanism for coordinating the intersectoral implementation of the recommendations of the United Nations High-Level Commission on Health Employment and Economic Growth, supporting WHO’s Global Strategy on HRH: Workforce 2030 and advancing universal health coverage (UHC). It gave impetus to two recommendations related to HRH data: 1) the establishment of an interagency data exchange and an online knowledge platform on the health and social service workforce; and 2) the establishment of an international platform on health worker mobility
May 2021	**WHA A74/8** Strengthening health information systems [9]	Measures progress made toward implementing WHA60.27 and given COVID-19 highlights the importance of data and health information systems (HIS) in guiding policy responses to the crisis; current data and HIS are inadequate to track health emergency protection, preparedness, and recovery
May 2021	**WHA 74.14**, Protecting, safeguarding, and investing in the health and care workforce [10]	The Working for Health 2022–2030 Action Plan [11] responds to WHA 74.14, which calls for a clear set of actions for accelerating investments in health worker education, skills, employment, safeguarding, and protection to 2030

### Step 2: Assembling the evidence

We examined the evidence gleaned through a desk review of targeted peer-reviewed and gray literature in the English language over the years 2000–2022 based on expert knowledge from the WHO Health Workforce department and the authors. Select interviews with subject matter experts at the WHO Health Workforce Department were conducted to capture information about HRHIS and for specific country case studies. A focused review of the gray literature was warranted, since development partners and ministries of health and other national public and private organizations have produced credible reports related to HRHIS that are not incorporated in peer-reviewed papers. Data from these sources were thematically analyzed and presented chronologically along with five country case studies covering various WHO regions and representing different types of successful implementations and financial and technical support. The authors applied a qualitative, iterative approach producing an in-depth analysis of empirical facts and related insights and identified new themes and inter-relationships, such as the relationship between epidemic/pandemic responses and the focus on HRHIS tool development. Some of the key themes that emerged from this iterative approach included: the chronology of HRHIS development, motivation for the development of HRHIS tools, the types of HRHIS tools or software and whether produced through government-led or donor-led development, type of country leadership (ministry of health, interministerial or multisectoral) and governance for HRHIS, impetus for donor investment, standardized indicators developed for global comparison, and the use of the HRHIS data for answering HRH policy, planning, and management questions.

### Step 3: Constructing the pathways, phases, and alternatives in developing and strengthening HRHIS

Table 2 summarizes a selective policy tracing and historical timeline over which major HRHIS decisions, policies, and initiatives occurred between 2000 and 2022.

**Table 2:** Policy tracing and historical timeline of HRHIS-related development, initiatives, and key milestones (2000–2022)

Before 2000 Pre-formation Phase	2000–2004 Formation Phase	2005–2010 Inception Phase	2011–2015 Evaluation Phase	2016–2022 Alignment Phase
Alma Ata Declaration (1978) of Health for All	WHO Rapid Assessment of Human Resources for Health (2004)	Management Sciences for Health (MSH) Human Resources Management Rapid Assessment Tool (2005)	Second Global Forum on HRH in Bangkok (2011)	HRH2030 (2016–2021)—USAID/PEPFAR global HRH project
Global Observatory for HRH data (1990)	Joint Learning Initiative Report: Overcoming the Crisis (2004)	Capacity and Capacity*Plus* (2004–2015), first USAID global HRH projects; developed and established iHRIS (open-source, web-based standards); launched HRH Action Framework (2009)	Third Global Forum on HRH in Recife (2013); 68 countries vow to improve HRH	Global Strategy on HRH: Workforce 2030 (WHA 69.19—2016), which accelerated results for NHWA and report on Global Health Observatory
WISN developed (1998)	PEPFAR launched in 2003; limited effectiveness without data about health workers, leading to the first global HRH project	World Health Report 2006 describes crisis in health workforce	WHA 67 adapted Recife Declaration (May 2014)	High-Level Commission on Health Employment and Economic Group Report (UN High Commission Report on investing in health workers and need for information, 2016)
		First Global Forum on HRH in Kampala (2008)		Fourth Global Forum on HRH in Dublin (2017)—launch of the NHWA
		DHIS2 first release, 2008 by HISP; open source, web-based for service statistics		Working for Health 2017–2021 Action Plan
				Working for Health 2022–2030 Action Plan

#### Precursors to HRHIS strategic investments (the formation phase, prior to 2005)

Prior to 2004, there were limited developments in HRHIS but there were precursors to the need for future investment in HRH data. The Alma Ata declaration in 1978 emphasized “Health for All” with a focus on primary health care [12] and the global monitoring of the Millennium Development Goals (MDGs) led to the creation of the WHO Global Health Observatory for country-level health data.

In 1998, the Workload Indicators of Staffing Need (WISN) method was first published, giving managers and planners a practical, evidence-based approach to estimate health workforce requirements based on actual workload [13]. However, the initial WISN software was not launched until 2010 [14].

Diallo et al. (2003) were among the earliest to describe potential HRH data sources, analytic approaches, and key indicators to assess HRH at country level and internationally. Various data sources existed for HRH (e.g., census surveys, registries) but the comparability and completeness of data varied within countries and indicators were not standardized for comparison across countries. Two key findings from this paper led to recommendations for substantial financial and technical investments in subsequent years. The first was the need to map health workers to internationally standardized classifications for health occupations, such as the four-digit international classification of occupations (ISCO) codes developed by the International Labor Organization, to allow for comparison of occupations across countries. Second was the need to develop HRHIS to address the limited, incomplete nature of HRH analyses, because “few available sources have been designed with the sole intention of producing information on HRH” [15].

#### Strategic investments to address the HIV/AIDS epidemic support foundational tools aligning with other global health workforce tools (inception phase, 2005–2010)

The earliest focus of international health development aid on strengthening HRHIS came from the US President’s Emergency Plan for AIDS Relief (PEPFAR), when service delivery efforts were hampered by the lack of data regarding the location, types, and numbers of health workers and those needing training. The USAID Capacity Project, launched in 2004 and led by IntraHealth International, became the first global HRH project focusing on supporting countries to track their health workers using credible information systems. Soon after the start of the Capacity Project, the World Health Report [16] indicated that not only did countries not know about the specifics of their health workforce but there was a significant shortage of doctors, nurses, and midwives, especially in sub-Saharan Africa. The Capacity Project developed the open source iHRIS software to support countries to track their registered and deployed health workforce and by 2009 nine countries had implemented iHRIS, mostly in Africa [17]. IntraHealth reports that there are more than 25 countries currently using iHRIS [18].

By the end of the Capacity Project in 2009, the Global Health Workforce Alliance had worked with the project to launch and pilot the HRH Action Framework [19], a precursor to the future PEPFAR HRIS Assessment Framework (2015), which is used to assess the maturity of HRHIS. Each functional area of HRHIS is assessed and scored (e.g., pre-service education, registration and licensing, health worker registry) according to its maturity from a paper-based system to a best practice web-based interoperable system using global standards [20].

During this same time frame, the Health Information System Programme (HISP) and global collaborators at the University of Oslo launched the district health information software (DHIS) in 2008, building on a concept first piloted in South Africa in the 1990s. Over 73 LMICs now use this system for collecting and analyzing health data. The updated DHIS2 uses open-source Java technology and is available through online applications free of charge as a global public good. DHIS2 and HRHIS are “book-ends” in that HRHIS provides HRH information by health facility and demographic details and DHIS2 offers facility-level service data to show the number of clients served by a specific facility [21]. Having these two information systems operating at the same facility allows managers to estimate in real time the number and type of health workers required by cadre according to the actual workload.

#### Data collected but not standardized nor strategically used; solutions emerge to align HRHIS data (evaluation phase, 2010–2015)

An outcome of the First Global Forum on HRH in Uganda in 2008, the Kampala Declaration called on countries to create health information systems and develop capacity for data management for evidence-based decisions [22]. Between 2010 and 2015 countries started to have the infrastructure and trained staff in information technology and data analysis, resulting in new technologies for HRHIS. In addition to iHRIS and DHIS2, these systems included professional council registries, personnel and payroll, master facility lists, national ID, performance management, pre-service education, and in-service training. These systems spanned ministries of health, finance, public service, local government, and professional councils, yet often lacked leadership and governance policies for data sharing, privacy, interoperability, and indicator standards, leaving countries still struggling to have an accurate picture of their health workforce [23, 24]. Countries such as Mali, Oman, and Tanzania [24–26] emerged as HRHIS implementers with limited support needed from international health development partners. In contrast, others previously reliant on implementing partners for support no longer continued to use and make improvements in their HRHIS once donor support ended or required support from other donors, such as in Uganda and Nigeria [23].

Riley et al. (2012) found a number of bottlenecks to HRHIS support for evidence-based human resources policy and planning such as lack of standardized processes for data collection, management and use, and limits on the availability and quality of the data for use by key stakeholders to support effective, efficient HRH strategies and investments at national, regional, and global levels [27]. Over and above that, the lack of evaluative research about HRHIS left questions about the quality, capabilities, and sustainability of these systems [28].

A recent WHO evaluation of HRHIS development from 2010 to 2020 showcased 12 LMICs that have adopted an HRHIS with various successes and challenges in use of the data [25]. In Kenya, timely and accurate workforce information and payroll efficiency led to improved planning and management and reduction in staff being paid who were not actually working (“ghost workers”). In Mali, HRHIS identified needs and allowed decision-makers to better plan and distribute health workers. Some critical success factors included: (1) adopting a systematic health systems strengthening approach; (2) strong leadership by the Ministry of Health (MOH); (3) pre-planning of cost absorption to sustain the HRHIS; (4) South-to-South collaboration [25, 29, 30]; (5) stakeholders’ involvement [24, 30]; (6) using a data warehouse approach (bringing multiple systems together, with local experts for developing software applications) [23]; and (7) reliable technical support to users.

Some of the key barriers to successful HRHIS implementation included: (1) lack of capacity and competencies in managing software; (2) infrastructure challenges (such as lack of network and computer storage capacities, lack of data back-up facilities, frequent power supply interruptions); (3) weak capacity in using information for operational and strategic planning needs at the subnational level; and (4) interoperability challenges between systems that are not addressed or are met with passive resistance from stakeholders [23, 25, 32, 33].

As HRHIS development evolved, other intermediary approaches were taking shape that were conducive to HRH data generation and use, such as WHO’s advancement of the Workload Indicators of Staffing Need method. WISN is used by countries to develop evidence-based facility- and cadre-level workforce requirements based on actual workload. This method has been used to equitably distribute health workers [14]. A special supplement of *Human Resources for Health* features 17 examples that demonstrate WISN implementation approaches, results, applications, and lessons learned from many WHO regions—including an application of WISN for COVID-19 [34, 35].

The Ebola epidemic in West Africa prompted the rapid development of a supplemental DHIS2 module for Ebola in 2014 to track individual Ebola patients and their contacts. Previously, DHIS2 only tracked aggregated clients. Three years before the Ebola outbreak, Liberia was using both DHIS2 and iHRIS so these systems were in place for urgent health workforce and health service management during the unanticipated Ebola epidemic, and improved internet availability strengthened the flow of coordinated standardized information instead of paper files [36].

#### The NHWA system strengthening approach to harness HRHIS development (alignment phase, 2016–2022)

Until the NHWA emerged in 2017 there were limited data standards in HRHIS. Health workforce information data became available that were not comparable across individual or multiple countries. Data collated by NHWA were used in the first State of the World’s Nursing Report (2020) and the State of the World’s Midwifery Report (2021). These reports provide up-to-date evidence on the number and requirements for nurses and midwives in 191 WHO member states and outline the contributions that nurses and midwives make for delivering the Sustainable Development Goals (SDGs) and UHC [37, 38].

Through the annual reporting by WHO member states and complementary data mining efforts, the NHWA data strengthen the generation of HRH data for monitoring and accountability in the implementation of national and regional health plans and strategies and allow countries to apply the latest methods for health workforce planning [36] and the global HRH strategy [40].

The NHWA implementation uses a system strengthening approach that is aligned with the WHO health labor market framework. It promotes quality and country leadership, and encourages evidence-based decisions, advocacy, and accountability. Although there are various approaches to implement the NHWA at country-level, there is an underlying “DNA” essential for success, which includes:

- An inclusive multisector governance mechanism
- Diversification of data sources
- System strengthening approach
- Countries’ needs and interests first
- Policy-driven data collection and use
- Partnership for improving health workforce data and evidence.

To ensure global access to NHWA, WHO developed a handbook, an implementation guide, and an online platform and web portal. Country focal points were appointed, and various national and regional trainings conducted to establish the country-level support necessary to launch and use NHWA results. NHWA consists of 78 standards-based indicators (which overlay with the health labor market framework) from the best available evidence at the time. Each country is expected to start by identifying the indicators that are most important to their health system and policy and management priorities.

When implemented well, NHWA provides in-depth information on the distribution, size, age, and characteristics of the health workforce, including workforce and service coverage, training, and financial resources. Identifying key NHWA priorities and using the results to strengthen health workforce coverage and quality of care requires ongoing interministerial and intersectoral stakeholder collaboration [40].

### Step 4: Defining the criteria to compare and contrast the evidence—developing an HRHIS for the SDG era

The focus of HRHIS-related tools, including NHWA, centers around the importance of health and care workers’ role in both UHC and achieving SDG 3, “Good Health and Well-Being” [41]. Health and care workers are paramount to achieving these global goals and HRHIS supports the planning and management of these workers by identifying that a sufficient number of them are available, equitably distributed, competent, and delivering quality and acceptable health services to achieve UHC. The COVID-19 pandemic has amplified the need to have a real-time understanding of the relationship between health and care workers and addressing the global crisis while continuing to provide routine health services. During the pandemic most countries struggled to have accurate, timely, available data on the health workforce, which has caused significant interruptions in health service delivery and the goal of achieving UHC. Thus, the lack of quality, available, up-to-date HRHIS information made the planning and management of health and care workers nearly impossible, causing undo stress on health workers and gaps in services delivery [42].

As the world was grappling with the early stages of the COVID-19 pandemic in 2020, the Bill and Melinda Gates Foundation funded an HRHIS cross-sectional analysis of 21 LMIC countries with a view to use HRHIS to identify concrete opportunities to better design, plan, and manage the health workforce [23]. The results of the assessment illuminated two key vectors necessary for successful country-level HRHIS application—governance and digital foundations. We used these two vectors to analyze the data for the five country case studies in the next section of this paper. The data from the Gates Foundation-funded study revealed four broad categories of HRHIS across the countries assessed (Table 3):

- Strong governance foundations: overall governance for transparency and accountability in decisions; digital governance, including data protection policy and digital health strategy
- Strong digital foundations: level of maturity of HRHIS, including interoperability across subsystems (e.g., HRHIS, payroll, DHIS2)
- Dual foundations: countries with strong governance and HRHIS
- Dual gaps: countries that did not have strong governance or HRHIS [22].

**Table 3:** Governance and digital/HRHIS strengths in 21-country assessment

	Dual foundations	Governance foundation	Digital foundation	Dual gaps
Characteristics	-More mature HRHIS, interoperability -Good governance foundation in place	- Good governance foundation in place - Paper or nascent HRHIS - Digital HRHIS	- Digital HRHIS in place or transitioning to one - Weaker governance and leadership structures	- Low governance and leadership structures - Low digital adoption with partial HRHIS in place
Countries	India (Uttar Pradesh, Karnataka), Oman, South Africa	Burkina Faso, Dominican Republic, Ethiopia, Malawi, Namibia, Philippines, Senegal	Kenya, Mali, Mozambique, Nigeria, Tanzania, Uganda	Bangladesh, Democratic Republic of the Congo, Guatemala, South Sudan

Key findings from the Gates Foundation-funded study include the following:

- Community health worker and private sector HRHIS data are not available to some governments and this impedes decision-making and planning
- Professional councils should be a strong HRHIS data source, but they are often under-resourced and lack authority or capacity to enforce licensing
- HRH management requires high-level coordination among various ministries and sectors
- HRHIS design and implementation often did not meet user needs for routine data and/or key stakeholders did not have access to HRHIS data
- Performance management is not prioritized nor aligned to health system goals and objectives
- Interoperability with payroll as a subsystem is a goal but hard to achieve because of the sensitive nature of payroll data.

These findings are consistent with the findings of Riley et al. (2012), where limits on the availability and quality of the data for use by key stakeholders were noted as a significant bottleneck, limiting the use of HRH data [27]. The assessment also gives several overall suggestions for successful use of HRHIS for country-level policy, planning, and management, including:

- The HRHIS should be the established source for all sectors and cadres
- A unique health workforce ID is essential for interoperability across information systems; however, these individual data should be aggregated for confidentiality reasons
- The HRHIS should function to meet user needs for routine management and administration
- Data access policies need to enable decision-making but protect privacy. Data sharing and interoperability across different data sources is critical, especially payroll, HRHIS, DHIS2, and regulatory bodies [23].

#### Projecting the principal outcomes through country experiences

This section focuses on five countries, representing different WHO regions, that have demonstrated excellence and examples of best practices in areas of governance/leadership and/or digital strengths in HRHIS. The references used for each case study are identified at the beginning of each section (Tables 4, 5, 6, 7, 8).

**Table 4:** Key digital and governance features of HRHIS in Oman

Digital features	Governance/leadership
• 80–90% interoperable data between human resources, HIS, and payroll for public and private sectors • Unique ID for employees • International standards for indicators • Indicators cover pre-service education, payroll, licensing, attendance, retention, exit labor force, HIS, supplies, and health facilities • WISN used for equitable staffing based on actual workload • NHWA used for overall health planning, management, and policies	• MOH has a Director of Information Technology overseeing all electronic data systems • Oman Vision for Health 2050 is a strategic plan for HRHIS, the health system, and services • Oman uses HRH data at the highest levels of government for health system development • Oman Observatory Committee reviews HRH data, enters these data into NHWA, and uses data for policy, planning, management, and improvements in the HRHIS • HRHIS used across various ministries and private sector

**Table 5:** Key digital and governance features of HRHIS in Kenya

Digital solutions	Governance/leadership
• iHRIS Manage and iHRIS Train track human resources and training • Includes community health workers and volunteers • rHRIS is the regulatory HRIS at the health professional councils • DHIS2 tracks health management information • Integrated personnel and payroll database (IPPD) used for hiring personnel and paying staff • NHWA standards-based indicators track SDGs • WISN used to determine health workers required by facilities based on actual workload • iHRIS, rHRIS, DHIS2, IPPD interoperable at county level; rRHIS and iHRIS Train interoperable	• Key policies: Kenya Health Sector HRH Strategy; applying Third Global Forum on HRH commitments; guidelines for sharing specialists across counties; Health Act 2017 for eHealth and mHealth; Ministry of Labor guidelines for recruitment and hiring of health workers • HRH Unit and Director at county level • Master training program to train and coach management team in areas of HRH data analysis and decision-making, WISN, budgeting, planning, and forecasting • Work councils established to deal with workforce labor issues • HRH Stakeholder Coordination Forum with interministerial, intersectoral, and county members uses data from NHWA and is a model for creating a multi-stakeholder platform County HRH Maturation Evaluation Tool provides key markers in transition from donor funding

**Table 6:** Key digital and governance features of HRHIS in Indonesia

Digital solutions	Governance/leadership
• Started with PEPFAR HRIS Assessment Framework to assess key HRIS functions, stakeholders, data flows, and NHWA readiness; assessment identified gaps in data use and analysis and provided evidence for NHWA investment • Applied Principles for Digital Development • Interoperability between HRIS and DHIS2 • Incorporated web applications and DHIS2 dashboards COVID-19 DHIS2 dashboard used to redeploy and redistribute health workforce	• NHWA stakeholders ensured optimization of HRIS to build platform for dynamic data analytics • Policy mechanisms developed for data sharing, such as ONE Data policy • Integrated NHWA into routine operations • Sector-wide approach for HRHIS • NHWA stakeholders continually assessing how to make improvements in HRHIS and led to guided COVID-19 responses by identifying training institutions, professional associations, and private sector to support emergency response • NHWA stakeholders analyze data to make informed health workforce decisions to optimize UHC outcomes

**Table 7:** Key digital and governance features of HRHIS in the Philippines

Digital solutions	Governance/leadership
• Future HRHIS will be linked to the data standards for 15 priority NHWA indicators • WISN conducted as interim measure to redistribute primary care workers • NHWA populated with data for 15 priority indicators, although interoperable HRHIS not yet developed	• Leadership change led to two HRH policies that guided future HRH interventions: *National HRH Master Plan 2020–2040* and the *Universal Health Care Act of 2019* • Health labor market assessment showed ineffective and inefficient human resources spending and maldistributed health workers • NHWA roadmap developed • Interministerial and intersectoral support prioritized for country-wide sustainable solutions

**Table 8:** Key digital and governance features of HRHIS in Mozambique

Digital solutions	Governance/leadership
• eSIP-Saude (locally developed HRHIS) uses enterprise architecture. The system is working toward interoperability with health professional registries, payroll, master facility list, biometric proof of life, pre-service and in-service education, and the health management information system • Not all components are in place to update records when changes occur, such as payroll • Multiple data systems still exist • Performance management is still paper-based	• Governance policies directed focus for HRHIS *National Human Resources Development Plan for Health 2015–2026* • WHO Observatory platform is used for uploading NHWA data, analyzing the results, and making HRH recommendations • National and subnational leadership oversaw the development and implementation of eSIP-Saude • Lack of data use culture limits use of available data • Local ownership continues to improve

#### Oman

Oman is an example of a locally developed, locally funded, and advanced integrated HRHIS that feeds its data into the NHWA [43–49]. Oman has both strong governance/leadership and strong digital systems. Leaders regularly review and use the data, identify bottlenecks and gaps in the HRHIS, and make improvements in the system to address the gaps. During the COVID-19 pandemic the minister of health could use their mobile phone to track all COVID-19 patients, ventilators, supplies and staff, and have a current analysis of patients and resources [47].

While Oman has experienced some of the challenges described earlier, it had the governance and leadership maturity to assess the gaps and where the country wanted to go, and to thoughtfully move in that direction while using the available data to guide improvements.

##### Milestones to achieving a functioning HRHIS

- Established clear objectives and strategy for the Al Shifa management information system in 2004 [39].
- Currently Al Shifa’s version 3 + is a comprehensive, integrated HIS, including electronic medical records, assets, inventory, and human resources management. It includes the private sector.
- Al Shifa uses national/civil identification numbers as the unique ID for each patient.
- Future plans are to develop Al Shifa using open-source Java technology to provide patient history and clinical information.
- Al Shifa is compulsory in the MOH.
- The Mawred Human Resources Management System is used by 42 government entities and has five modules: human resources, payroll, self-service, electronic salaries transfers, and attendance.
- Data from Mawred, Al Shifa, and WISN are used to populate NHWA.

#### Kenya

Kenya is an example of a country that uses free open-source software with external donor support as well as a data warehouse approach for interoperable information systems for NHWA [40, 49–54]. Kenya has moderately strong HRH governance. Both governance and digital systems strengthened as they devolved to a county governance system beginning in 2013.

##### Milestones to achieving a functioning HRHIS

- Kenyan HRHIS date back nearly 20 years with the advent of one of the first regulatory HRIS in Africa as well as a nascent iHRIS for public sector health workers. Both HRHIS were initially developed with donor funding from CDC (regulatory HRIS) and USAID (iHRIS for public health workers).
- In 2013 the sudden devolution to county governments over a 6-month period instead of the requested 3 years caused an urgent need for governance, leadership, and standards-based interoperable information systems for evidence-based HRH decisions.

#### Indonesia

Indonesia is an example of a country that used NHWA as a starting point for its national HRHIS to identify priority indicators and steps of implementation and data gathering [55, 56]. Indonesia has strong governance and leadership capacities, which led to improvements in HIS and utilization of NHWA results.

##### Milestones to achieving a functioning HRHIS

- Indonesia followed the WHO recommendation to implement NHWA progressively, based on the availability of data, taking a multisectoral approach, and using existing evidence and systems with a focus on improving the availability of quality data to make evidence-based decisions.
- The NHWA, initiated through a joint mission by WHO, USAID, and the USAID HRH2030 Program, resulted in a landscape analysis of the stakeholders and information systems, leading to an implementation plan with prioritized indicators.

#### Philippines

The Philippines is another example, where the NHWA roadmap was used as a starting place for developing a national HRHIS [56–59]. Its experience represents a convergence of policy, leadership, technical support, and digital solutions.

##### Milestones to achieving a functioning HRHIS

- The Philippines had multiple standalone information systems developed from 2007 to 2009 scattered across the Department of Health (DOH) and other intersectoral and interministerial sites. The Philippines had a major challenge in data flow from the district to the central level. The data were not comparable across systems, fragmented in terms of data collection protocols, and data use policies were not in place resulting in lack of information about the number, cadre, and location of health workers.
- Leadership and governance changes at the DOH between 2016 and 2020 resulted in two key policies, *the National HRH Master Plan 2020–2040* and the *Universal Health Care Act of 2019.* Donor support has been available to advance sustainable HRH solutions.
- The DOH prioritized the health workforce as the backbone of a health care system that is accessible, affordable, accountable, and reliable.
- The DOH was supported by USAID and WHO to develop the process, procedures, and roadmap to guide implementation of NHWA starting with the NHWA roadmap. An NHWA mapping exercise on over 15 indicators was prioritized and data populated in the NHWA platform.
- As an interim measure, WISN was institutionalized at all levels of the health system for optimizing the number of health workers per cadre aimed at improving quality of primary health care delivery.
- Future HRHIS development would be standards-based using NHWA priorities, interoperable, usable across the country, and producing quality data available upon demand.

#### Mozambique

Mozambique is an example of a country that developed a local HRHIS with limited financial and technical support from donors [23, 24, 60]. Mozambique achieved the development and use of HRHIS with strong governance and leadership, although it still struggles with building a data-use culture for decision-making.

##### Milestones to achieving a functioning HRHIS

- Mozambique is an early adopter of NHWA as it was one of the three countries, where NHWA was originally piloted in 2016 with 42 out of the original 90 indicators from the pilot uploaded at that time.
- Key HRH policies directed the focus for health sector goals and objectives, including HRHIS.
- The HRHIS, called eSIP-Saude, was locally developed and has local ownership.
- NHWA was implemented through the HRH Observatory; HRH Observatory stakeholders are responsible for analyzing the NHWA data and making HRH policy recommendations.
- HRHIS only includes the public sector and does not cover the private sector nor community health workers.

### Step 6: Confronting the trade-offs and lessons learned from country experiences

We observed that different factors and enablers facilitated HRHIS development in each of the five countries. In the case of Oman, a successful interoperable HRHIS required both governance/leadership and digital solutions that take time to develop and are iterative. Multisectoral and interministerial stakeholders are required to build and use the available systems and the data they produce all the while improving the quality of both the information systems and their data.

In the case of Kenya, having strong interoperable digital systems with supporting governance, intersectoral leadership, and policies for data sharing and ownership allowed the country to identify retirees and those who have exited the workforce and use those resources to hire other staff, using the WISN method to identify where to place needed health workers.

Indonesia is a good example of a country that systematically applied the “DNA principles” of NHWA to achieve an interoperable NHWA—country-led and policy-driven, with multisectoral stakeholders, multiple data sources applied, integration of NHWA into routine operations, and a strong partnership to develop HRHIS. Applying the Principles for Digital Development led to an interoperable HRHIS that supports rapid analysis and visualization of health workforce-related data to respond to routine operations, such as maternal and child health outcomes and HIV/AIDS care and treatment, as well as a national multisectoral response to redistribute and redeploy health workers for an emergency, such as COVID-19.

Although the Philippines has not yet achieved an interoperable HRHIS to support its vision, the country demonstrates that using the NHWA implementation process and prioritizing indicators, it can begin to achieve usable data for evidence-based decisions before completing the NHWA process and having an interoperable HRHIS. In the interim the Philippines started by developing key policy documents, conducting a health labor market assessment, and rolling out the NHWA roadmap. By prioritizing the NHWA indicators and applying the available credible information on those indicators and the WISN facility-based results, the Philippines’ MOH can equitably redistribute the workforce.

Finally, Mozambique’s example demonstrates that having strong leadership and governance with a multisectoral observatory team of decision-makers and data producers resulted in a strategic plan and investments that led to a country-developed HRHIS for planning and managing the health workforce. The local ownership and policy environment promote making the needed improvements to HRHIS and using the available data.

### Step 7: A synthesis of the evidence

**Fig. 1:**
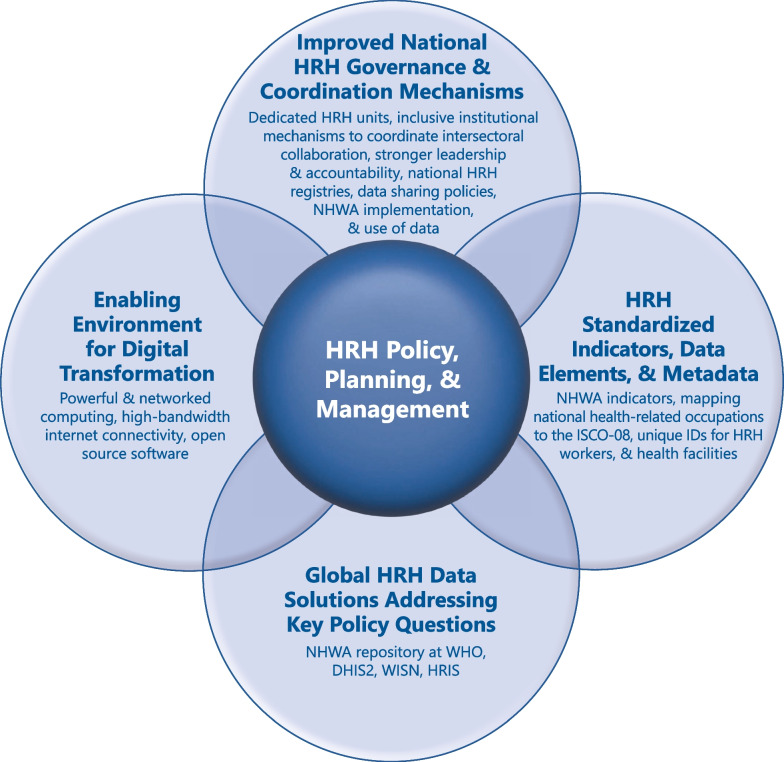
Two decades of HRH investment and innovation has resulted in four domains of inter-related global HRHIS advances to promote evidence-based HRH policy, planning, and management solutions

Figure 1 illustrates the high-level results of the policy-tracing trend analysis and how the evolving technical and financial investments have shaped the development of HRHIS over the last 20 years, particularly among LMICs. Our synthesis offers insight on how to sustain further improvements of the HRHIS and expand its configuration to future needs around building health systems preparedness and resilience, and the practicalities of integrating newer occupations and the specialized health workforce:

- Countries need to be supported to mature their HRHIS to meet their priority NHWA indicator requirements, using maturity assessment HRHIS tools to ensure they are applying current digital principles in making iterative country-driven improvements directed by a multisectoral, multidisciplinary, interministerial stakeholder leadership team. The HRHIS should cover the key HRHIS indicators, including education, registration, unique ID, deployment, retention, salary, professional development, and performance management, so managers do not need to access various data sources to manage the health workforce. This stakeholder leadership team should ensure that data are used for routine planning and management of the health and care workers.
- The usability of the WISN software can be greatly improved by making it web-based. Its implementation time can also be reduced by providing access to prioritized health service activities and activity standards across countries and regions.
- There should be a readily available module in DHIS2/WISN for health emergencies that can track individual clients and not just aggregate clients, building on the ones developed for the Ebola and COVID-19 pandemics. Countries should be able to manage essential health services and emergencies with the same HRHIS, as is the case in Oman and Indonesia.
- Partnerships such as the one with the University of Oslo (as a WHO collaborating center) for DHIS2 improvements could be a potential model to support various development partners, international agencies, and technical experts to test and refine NHWA, HRHIS, and WISN software improvements.
- HRHIS’ need to include activities such as performance management and attendance that contribute to assessing access to health services and quality of care, which are frequently not tracked in the current HRHIS. Cadres and sectors not included in most current HRHIS need to be integrated, such as pharmacy workers, laboratory workers, community health workers, primary care workers, including social services, and private and NGO sector health and care workers. This will provide a more holistic view of the workforce available and required to deliver UHC.
- Country-level leadership and governance need to ensure that policies for data sharing, interoperability, confidentiality, data storage, and data back-up, among others, are available so data are available and reliable for health workforce policy, planning, and management decisions.
- The next version of NHWA should ensure that country-level HRHIS data can be interoperable with NHWA by applying good governance digital principles, so that real-time data are available for policy development, planning, and management of the health and care workers. These real-time data are agile and available to manage routine health service delivery as well as improve planning and response in the face of public health emergencies and epidemics.

#### Limitations of this manuscript

This manuscript did not conduct an exhaustive review of the HRHIS literature and relied mainly on targeted gray literature and national reports for the case studies. It is possible that a broader examination of the literature and country-level in-depth interviews for the case studies could have yielded further results.

### Step 8: Conclusions, major influences, and future outlook

Over the past 20 years, there have been documented improvements in the availability of HRHIS data for policy, planning, and management. The policy-tracing analysis conducted in this study demonstrates the serial progression in global mandates and how those have gradually influenced country commitments and actions in strengthening HRHIS, coupled with increased investments in financial and technical resources. Successful HRHIS development takes time and iterative improvements to mature the governance and digital-related elements necessary for a well-functioning HRHIS. In the context of developing HRHIS, the NHWA approach offers a country-specific roadmap for NHWA implementation to meet fundamental HRH data needs and the use of that data for decision-making. NHWA also provides a mechanism for cross-country HRHIS maturity comparisons.

Today and into the future, what elements describe a well-functioning HRHIS? “A canonical source of truth for health workers and their location in all sectors and cadres; a unique ID to link data to unique health workers and facilitate interoperability; functionality that meets user needs for routine management and administrative tasks especially at the subnational level; data access that enables decision-making but protects privacy and security needs; data sharing and interoperability across different HRH data sources, including payroll, HRIS, and facility registries. It is anchored on three pillars: governance and ownership; actor incentive structures; and system design matched to country context” [23].

The COVID-19 pandemic demonstrates the continuing necessity to strengthen HRHIS as a global public good to ensure that each country has fundamental data on and knowledge of its health and care workers (who are they, where are they available, and what services are they involved in. Having quality health and care workforce data available is a global and moral imperative to support countries’ preparedness and response before the next pandemic or emergency strikes.

## Data Availability

The references include all information used to develop the manuscript and its conclusions.

## Abbreviations

DHIS: District Health Information Software
HIS: Health information system
HRHIS: Human resources for health information system
JLI: Joint learning initiative
LMIC: Low- and middle-income country
MDGs: Millennium development goals
MOH: Ministry of Health
NHWA: National health workforce accounts
SDGs: Sustainable development goals
UHC: Universal health coverage
WHA: World health assembly
WHO: World Health Organization
WISN: Workload indicators of staffing need
