# Correction: The transition of human resources for health information systems from the MDGs into the SDGs and the post-pandemic era: reviewing the evidence from 2000 to 2022

**DOI:** 10.1186/s12960-024-00890-4

**Published:** 2024-01-11

**Authors:** Pamela A. McQuide, Andrew N. Brown, Khassoum Diallo, Amani Siyam

**Affiliations:** 1 Global Health Workforce Consultant, IntraHealth International, 6340 Quadrangle Drive, Suite 200, Chapel Hill, USA; 2 Global Health Workforce Consultant, Canberra, Australia; 3 Coordinator Data, Evidence and Knowledge Management UHL Division, World Health Organization, Geneva, Switzerland; 4 Health Information System, Regional Office for South-East Asia, World Health Organization, Geneva, Switzerland

**Correction: Human Resources for Health (2023) 21:93** 10.1186/s12960-023-00880-y

Following the publication of the original article [1], the authors identified that the subheading was incorrect. The correct subheading is given below.

The incorrect subheading is: Projecting the principal outcomes through country experiences

The correct subheading is: Step 5: Projecting the principal outcomes through country experiences

The original article [1] has been corrected.
